# Cashew apple extract inhibition of fat storage and insulin resistance in the diet-induced obesity mouse model

**DOI:** 10.1017/jns.2015.30

**Published:** 2015-12-02

**Authors:** Vickram Beejmohun, Cyril Mignon, Aude Mazollier, Marie Peytavy-Izard, Dominique Pallet, Manuel Dornier, Nicolas Chapal

**Affiliations:** 1 Dialpha SAS, Parc Agropolis 2, 2196 boulevard de la Lironde, F-34980 Montferrier sur Lez, France; 2 CIRAD (French Agricultural Research Centre for International Development), UMR95 QualiSud, TA B95/16, 73 rue Jean-François Breton, F-34398 Montpellier cedex 5, France; 3 Montpellier SupAgro, UMR95 QualiSud, B.P. 5098, 1101 avenue Agropolis, F-34093 Montpellier cedex 5, France

**Keywords:** Cashew apple extract, Obesity, Pre-diabetes, Metabolic syndrome, CAE, cashew apple extract (Cashewin^™^), DIO, diet-induced obesity, HFD, high-fat diet, HOMA-IR, homeostasis model assessment-insulin resistance, ND, normal diet, PEAT, peri-epididymal adipose tissue

## Abstract

The cashew apple is an unvalued by-product from the cashew nut industry, of which millions of tonnes are simply discarded globally. Interestingly, however, cashew apple nutrients may have beneficial effects for health even if these are still poorly described. The present study was designed to evaluate the effect of a hydro-alcoholic extract of cashew apple (cashew apple extract; CAE; Cashewin^™^) on obesity and diabetes, in two experimental designs using the diet-induced obesity (DIO) mouse model. First, in the preventive design, mice were treated orally with the CAE at the dose of 200 mg/kg body weight from the first day under a high-fat diet (HFD) and during 8 weeks thereafter. Second, in the curative design, the animals were first maintained under the HFD for 4 weeks and then treated with the CAE for a further 4 weeks under the same regimen. For both experimental designs, body weight, peri-epididymal adipose tissue, liver weight, food consumption, glycaemia, insulinaemia and insulin resistance were assessed. In both designs, the CAE significantly reduced body-weight gain and fat storage in both the peri-epididymal adipose tissue and the liver for mice under the HFD. This was achieved without modifying their energy consumption. Furthermore, glycaemia, insulinaemia and insulin resistance (homeostasis model assessment-insulin resistance) of the DIO mice were significantly lowered compared with the control group. Thus, a well-designed hydro-alcoholic extract of cashew apple could provide an attractive nutritional food ingredient to help support the management of body weight and associated metabolic parameters such as blood glucose and insulin levels.

According to the WHO, obesity and overweight are considered to be the fifth largest risk factor for global deaths. The number of obese people has nearly doubled since 1980 and the WHO estimated in 2014 that more than 1·9 billion adults over 18 years of age were overweight, with more than 600 million being obese^(^^1^^)^. Scientific publications have showed that overweight is one of the major risks factors for metabolic syndrome disorders. Elevated BMI (>30 kg/m^2^) increases the risk of developing type 2 diabetes, hypertension, cholesterol abnormalities, CVD, Alzheimer's disease and inflammation-based pathologies^(^^2^^–^^5^^)^.

In the struggle to manage body weight, different therapeutic approaches have been investigated. Synthetic anti-obesity drugs have emerged, each having their own effects on fat absorption and metabolism, but all showing adverse effects on humans^(^^6^^,^^7^^)^. For example, Orlistat (tetrahydrolipstatin) is the only gastrointestinal lipase inhibitor drug approved by the Food and Drug Administration and the European Agency for treating people suffering from obesity and hypercholesterolaemia, even if it causes some gastrointestinal side effects^(^^8^^–^^11^^)^. Consequently, alternative nutritional approaches have been investigated and are gaining scientific and public interest^(^^2^^,^^12^^,^^13^^)^.

Cashew trees (*Anacardium occidentale* L.), while native to Brazil, are now widely spread across many tropical countries. They are mainly cultivated for their nuts, the ‘true fruit’, which is considered to be the main economic product. However, along with the nuts, a pseudo fruit is also collected, known as cashew apple and weighing around 90 % of the harvested mass^(^^14^^)^. Cashew apples are considered to be a by-product of the cashew nut industry. The ripe apples, although known to be edible, are not widely appreciated by consumers because of their limited shelf life as well as their sour and astringent taste^(^^15^^,^^16^^)^. Several industries have tried to develop suitable processes for giving a more widely accepted taste to this fruit either in juice^(^^15^^)^ or wine^(^^17^^)^ forms. Currently only 12 % of Brazilian cashew apples are being processed for food or beverage, with the remaining apples being either left on the ground to rot or used for animal feed^(^^18^^,^^19^^)^.

Few publications have reported the phytochemical constituents of the cashew apple^(^^20^^–^^23^^)^. Michodjehoun-Mestres *et al*. have identified and quantified several glycosylated flavonols in the cashew apple^(^^20^^)^ next to a new cinnamic acid derivative called 1-*O-trans* cinnamoyl-*β*-d-glucopyranose^(^^22^^)^. To date, some literature has discussed the hypoglycaemic properties of extracts obtained either from cashew tree stem-barks^(^^24^^–^^26^^)^, leaves^(^^27^^–^^29^^)^ or nuts^(^^30^^)^ in animal models. Prasertsri *et al*. suggested in a clinical study that cashew apple juice supplementation increased fat utilisation during high-intensity exercise in both trained and untrained subjects, yet with no observed effects on metabolic profiles^(^^31^^)^. To our knowledge, however, no data presenting the effect of cashew apple extracts (CAE) on metabolic syndrome parameters have been published to date.

The present study was carried out to evaluate the efficacy of a specific CAE (Cashewin^™^) on C57BL/6 diet-induced obesity (DIO) mice in two different experimental designs to assess the preventive and curative effects on the reduction of body-weight gain and fat storage, hyperglycaemia, hyperinsulinaemia and insulin resistance in an animal model of the metabolic syndrome.

## Methods

### Reagents

Chemical standards and other analytical grade reagents and solvents were from Sigma-Aldrich Chemical Co. and Extrasynthese.

### Cashew apple extract

The CAE (Cashewin^™^) tested in this study is manufactured by Dialpha. It is a hydro-alcoholic extract of cashew apple residues (*Anacardium occidentale* L.). Cashew apples were crushed and pressed to remove their juice. The press cake was used to prepare the extract. A quantity of 350 g of the press cake was extracted with 3·5 litres of a 50:50 water–ethanol solution (v/v) for 2 h at 50°C. After a first solid–liquid separation, the solid residue was extracted a second time in the same conditions with 2·45 litres of solvent. The grouped filtrates were concentrated at 50°C in a rotary evaporator and then converted into powder by freeze-drying. The average yield of extraction was about 5 % from the cashew apple residues.

Phenolic constituents of CAE were measured by the colorimetric method using Folin–Ciocalteu reagent (ISO 14502-1) for its total phenolic content and by HPLC for the aromatic compounds.

Analytical HPLC analysis of the CAE was performed on an Ultimate 3000 Dionex system fitted with a reversed phase C-18 column (250 × 4·6 mm internal diameter × 5 μm; ACE^®^) and with a guard column, operated at 30°C. The mobile phases used were 0·1 % formic acid in high-purity water (solvent A) and acetonitrile (solvent B), utilising the gradient as shown in Table 1 over a total run of 86 min with a flow rate of 0·7 ml/min. Samples were prepared at a concentration of 10 g/l in 80 % (v/v) methanol and injected at a level of 20 μl. Aromatic compounds in the extract were detected at 280 nm.

**Table 1.** HPLC gradient system program

Time (min)	Solvent A (acidified water)	Solvent B (acetonitrile)
0	95	5
10	90	10
20	90	10
40	80	20
50	80	20
65	70	30
75	50	50
76	0	100
85	0	100
86	95	5

The identification of phenol constituents in the CAE by HPLC was based on the literature^(^^20^^,^^22^^)^. The main flavonoids, i.e. myricetin and quercetin derivatives, were quantified using their aglycone moieties as standards. 1-*O-trans*-cinnamoyl-β-d-glucopyranose was quantified using *trans*-cinnamic acid as standard.

The analysis of other CAE constituents was subcontracted to a specialised analytical laboratory, where humidity (NF V04-401), proteins, lipids, ashes and total carbohydrate (ARR 08/09/1977) were measured.

### Animals and experimental design

Male C57BL/6NCrl mice aged 5 weeks old weighing about 20 g were purchased from the Charles River Laboratories. Three mice were housed per cage. The animal room environment was controlled with a temperature of 22 ± 2°C, 60 % humidity, and day–night cycles of 12 h light–12 h dark (19·00–07·00 hours). Animals were allowed to acclimatise to the laboratory environment for 8 d under a normal diet (ND; EF R/M Control, ref. E15000; Ssniff; see Table 2). The protocols were approved by the following ethics committee and recorded on 23 April 2012 under the reference CEEA-LR-12010: ‘Comité Régional d'Ethique sur l'Expérimentation Animale’, Pharmacology Laboratory, Pharmacy University, 15 Avenue Charles Flahault, 34093 Montpellier, France.

**Table 2.** Composition of the normal diet (ND) and the high-fat diet (HFD)

	ND	HFD
Energy		
Energy from proteins (%)	23	17
Energy from carbohydrates (%)	66	29
Energy from fat (%)	11	54
Gross energy (kJ/g)	18·0	24·0
Metabolisable energy (kJ/g)*	15·0	20·9
Crude nutrients		
Proteins (%)	20·8	20·7
Starch (%)	46·8	16·9
Sugar (%)	10·8	16·6
Fibre (%)	5·0	5·0
Fat (tallow) (%)	4·2	30·7
Saturated fat (%)	0·7	14·9
Monounsaturated fat (%)	1·1	12·4
Polyunsaturated fat (%)	2·4	1·4
Cholesterol (%)	–	0·009

* Metabolisable energy was calculated with the Atwater factors.

The first study evaluated the preventive effect of chronic oral administration during 8 weeks of the CAE in the C57BL/6 DIO mice. After the acclimatisation period, mice were randomly assigned to the three different study groups according to their body-weight values in order to obtain homogeneous groups (nine animals per group). The ND-control group was kept under the ND and treated with the vehicle (Milli-Q water), the HFD-control group was switched to high-fat diet (HFD; EF R/M with 30 % fat, ref. E15126; Ssniff; see Table 2) and treated with the vehicle, and the HFD-CAE group was switched to the HFD and treated with the CAE at the dose of 200 mg/kg body weight. The extrapolation of this animal dose to human equivalent dose was based on the body surface area (Guidance for Industry – Estimating the Maximum Safe Starting Dose in Initial Clinical Trials for Therapeutics in Adult Healthy Volunteers; http://www.fda.gov/downloads/Drugs/.../Guidances/UCM078932.pdf) normalisation method and resulted in a dose of 1200 mg CAE for a 70 kg person. This corresponded to a consumption of around 200 g of cashew apple, i.e. two to three fruits daily.

The CAE prepared in solution in Milli-Q water or the vehicle (Milli-Q water) were given by oral administration every morning between 08·00 and 10·00 hours. The dose volume was 10 ml/kg body weight and the actual volumes administered were calculated and adjusted based on the most recent body weight of each animal. The diets and filtered tap water were provided *ad libitum*.

The second study assessed the curative effect of chronic oral administration during 4 weeks of the CAE in the C57BL/6 DIO mice. After the acclimatisation period, mice were first submitted to a HFD for 4 weeks to induce obesity and pre-diabetes. The ND-control group was kept under the ND. At the end of this induction period, mice were randomly assigned to the two remaining study groups according to their body-weight values in order to obtain homogeneous groups (nine animals per group). The ND-control group was kept under the ND and treated with the vehicle (Milli-Q water), the HFD-control group was kept under HFD and treated with the vehicle, and the HFD-CAE group was kept under the HFD and treated with the CAE at the dose of 200 mg/kg body weight.

In the two studies, mice were weighed three times per week and food consumption was monitored per cage once per week. Glycaemia of the mice was measured weekly in animals which had fasted for 5 h. One drop of blood was collected via the tail vein for glucose determination using a hand-held glucometer (OneTouch Ultra 2; LifeScan). At the end of the study, after a 5 h fast, animals were anaesthetised by an intraperitoneal injection of a mix of ketamin (Imalgen^®^ 1000) and xylazine (Rompun^®^ 2 %). A terminal blood sample was collected via cardiac puncture using heparin as anticoagulant for plasma preparation. This blood sampling resulted in the death of the animals. The peri-epididymal adipose tissue (PEAT) and the liver were harvested to measure their weights. Fasting plasma insulin levels were measured using an ELISA kit (Mercodia). Then the insulin resistance index (homeostasis model assessment-insulin resistance; HOMA-IR) was calculated using equation (1):
1
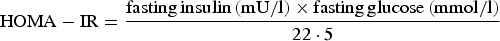


The liver TAG content was measured in the preventive design. The livers were homogenised in a mixture of chloroform–methanol (2/1, v/v) and lipids were extracted using the Folch method^(^^32^^)^. The TAG content was determined using the respective ELITech kits (TGML-0250 and CALI-0550; ELITech).

### Statistical analysis

Results were expressed as means and standard deviations (*n* 9). The statistical analysis was conducted using Microsoft Excel software. One-way or repeated ANOVA tests as well as the Fisher's least square difference *post hoc* test were executed. Values of *P* < 0·05 were considered statistically significant when comparing two different groups.

## Results

### Cashew apple extract

The results obtained showed that the CAE was composed of about 5 % total polyphenols, 13 % humidity, 7 % proteins, 7 % lipids, 3 % minerals and 65 % total carbohydrates.

**Fig. 1.**
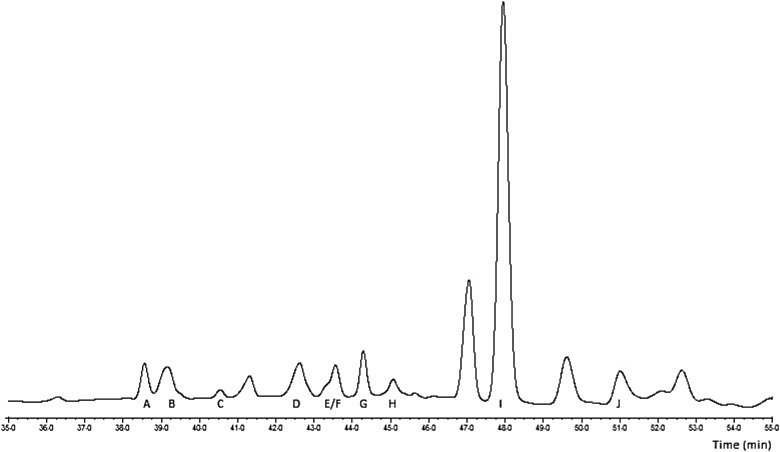
HPLC chromatogram of the cashew apple extract (35 to 55 min segment) at 280 nm. Peak A: myricetin 3-*O*-galactoside; peak B: myricetin 3-*O*-glucoside; peak C: myricetin 3-*O*-xylo-pyranoside; peak D: myricetin 3-*O*-arabino-pyranoside; peak E: myricetin 3-*O*-arabino-furanoside; peak F: myricetin 3-*O*-rhamnoside;peak G: quercetin 3-*O*-galactoside; peak H: quercetin 3-*O*-glucoside; peak I: 1-*O-trans*-cinnamoyl-β-d-glucopyranose; peak J: quercetin 3-*O*-rhamnoside.

The HPLC profile for aromatic molecules is presented in Fig. 1, where peaks A to F represent the myricetin derivatives, peaks G, H and J the quercetin derivatives, and peak I the cinnamic acid derivative. The phytochemical content of the CAE expressed in g/kg dry extract was 2·7 g myricetin derivatives, 1·2 g quercetin derivatives and 1·4 g 1-*O-trans*-cinnamoyl-β-d-glucopyranose.

### Preventive effects of the cashew apple extract on diet-induced obesity mice

#### Preventive effects of the cashew apple extract on body weight, fat storage and energy consumption

**Fig. 2.**
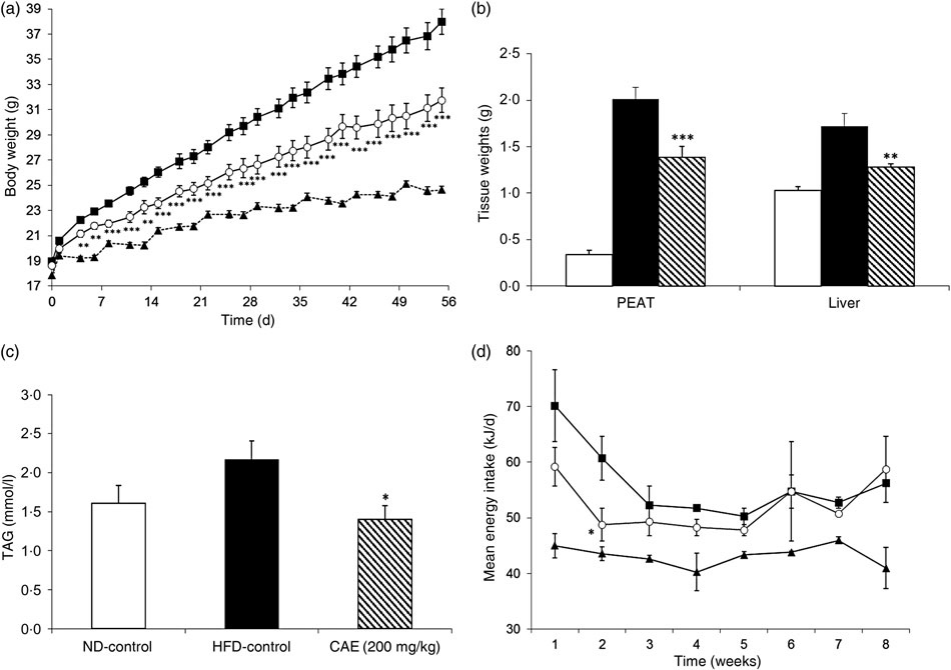
Preventive effects of the cashew apple extract (CAE) on (a) body weight, (b) peri-epididymal adipose tissue (PEAT) and liver weights, (c) liver TAG content and (d) energy intake in diet-induced obesity mice. (a) and (c): --▲--, Normal diet-control (ND-control); –■–, high-fat diet-control (HFD-control); –○–, CAE (200 mg/kg body weight). (b) and (c): □, ND-control; ■, HFD-control; \\\, CAE (200 mg/kg body weight). Values are means (*n* 9), with standard errors represented by vertical bars. Mean value was significantly different from that of the HFD-control group: * *P* < 0·05, ** *P* < 0·01, *** *P* < 0·001.

Mice under the HFD (HFD-control) gained much more weight than those under the ND (ND-control; Fig. 2(a)). The difference in body weight between the two groups became statistically significant from the fourth day under the diets. At the end of the study, after 8 weeks of treatment, the HFD-control group reached an average body weight of 38 (sd 1) g, whereas the ND-control group reached an average weight of 24·6 (sd 0·3) g. Thus, the HFD-control group gained almost three times more weight than the ND-control group: 19 (sd 0·92) g for HFD *v.* 6·8 (sd 0·32) g for ND mice. This increase in body weight induced by the diet is primarily due to the storage of energy under the form of perivisceral fat. Indeed, PEAT weights of mice under the HFD were multiplied by six during the study compared with those of mice under the ND: 2·01 (sd 0·13) and 0·34 (sd 0·05) g for the HFD and ND, respectively (Fig. 2(b)). Next to that, the HFD also induced the storage of fat in the liver (hepatic steatosis). The colour of the liver became white instead of red for mice under the ND and the liver weight was increased by 67 %: 1·72 (sd 0·14) g for HFD *v.* 1·03 (sd 0·04) g for ND mice (Fig. 2(b)). Furthermore, the liver TAG level was increased by 34·2 % in the HFD-control group: 2·16 (sd 0·24) mmol/l for HFD *v.* 1·61 (sd 0·22) mmol/l for ND mice (Fig. 2(c)). Combined, these results show that the HFD containing 30 % fat with long-chain SFA (beef tallow) induced a well-established model of obesity and related disorders.

As shown in Fig. 2(a), the CAE at the dose of 200 mg/kg body weight reduced by almost half the body-weight gain induced by the HFD (48·4 % at the end of the study). This effect was statistically significant from the fourth day of treatment (*P* < 0·01) and increased all along the study. Mice under the HFD treated with the CAE gained 13·1 (sd 1·09) g, while HFD-control mice treated with water gained 19 (sd 0·92) g and ND-control mice treated with water gained 6·8 (sd 0·32) g. This reduction in body-weight gain is at least partly due to a reduction of fat storage in the perivisceral fat. Indeed, the CAE reduced by 37·2 % fat storage into PEAT induced by the HFD (*P* < 0·01). PEAT weight of CAE-treated mice was 1·39 (sd 0·11) g, compared with 2·01 (sd 0·13) and 0·34 (sd 0·05) g for HFD-control mice and ND-control mice, respectively (Fig. 2(b)). Also, the CAE reduced fat storage induced by the HFD in the liver by 63·8 % (*P* < 0·01). Liver weight of CAE-treated mice was 1·28 (sd 0·04) g, compared with 1·72 (sd 0·14) and 1·03 (sd 0·04) g for HFD-control mice and ND-control mice, respectively (Fig. 2(b)). This reduction in liver weight was correlated with a significant reduction in liver TAG content of 138 % (*P* < 0·05). In fact, the liver TAG levels for the CAE-treated mice was 1·40 (sd 0·18) g, compared with 2·16 (sd 0·24) and 1·61 (sd 0·22) g for HFD-control mice and ND-control mice, respectively (Fig. 2(c)).

After a stabilisation period of 2 weeks, the energy intake of the different groups remained stable throughout the experimental duration. Animals under the HFD consumed more energy than those under the ND. No statistically significant difference in energy consumption was observed between the HFD-control group and the HFD-CAE group (Fig. 2(d)).

#### Preventive effects of the cashew apple extract on glycaemia, insulinaemia and insulin resistance

**Fig. 3.**
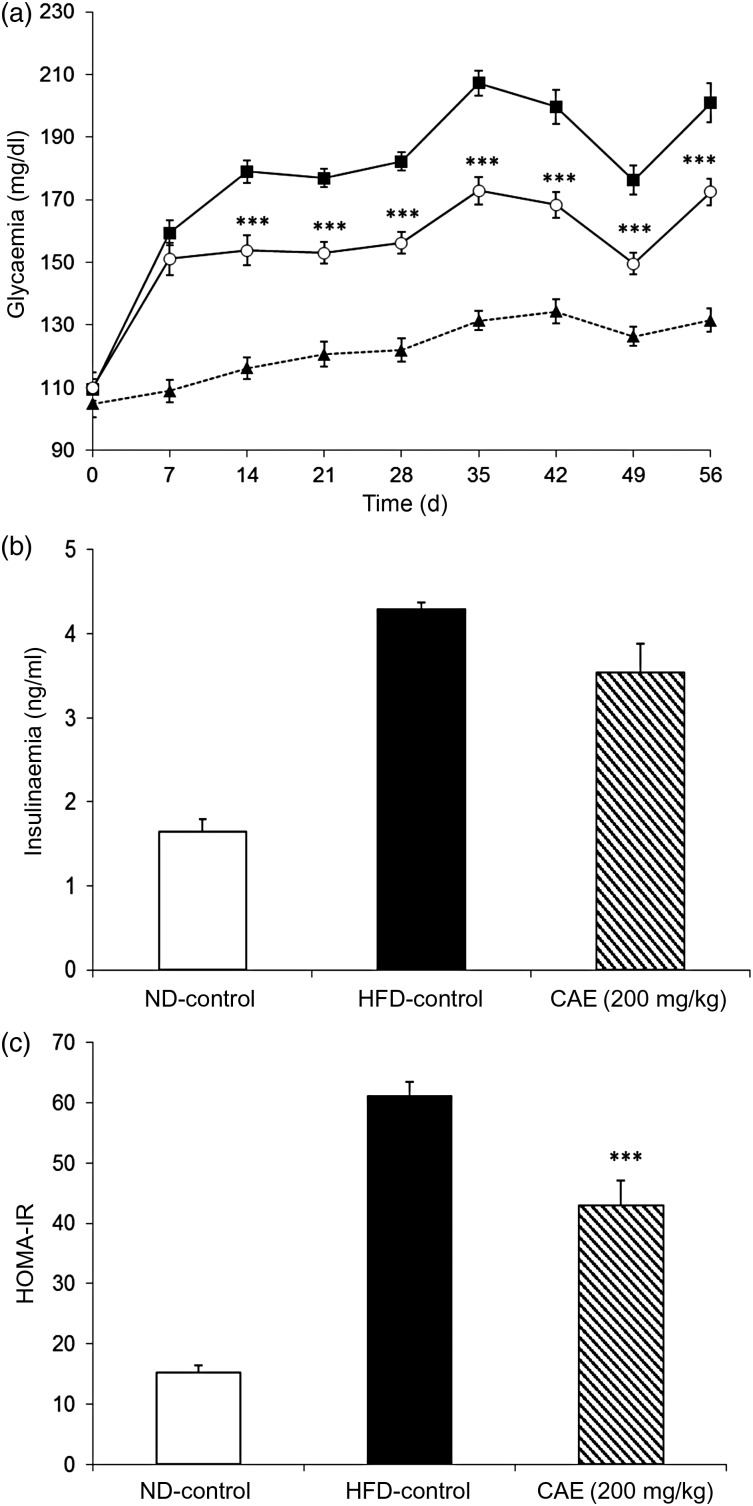
Preventive effect of the cashew apple extract (CAE) on (a) glycaemia, (b) insulinaemia and (c) insulin resistance (homeostasis model assessment-insulin resistance; HOMA-IR) in diet-induced obesity mice. (a): --▲--, Normal diet-control (ND-control); –■–, high-fat diet-control (HFD-control); –○–, CAE (200 mg/kg body weight). To convert glycaemia from mg/dl to mmol/l, multiply by 0·0555. Values are means (*n* 9), with standard errors represented by vertical bars. Mean value was significantly different from that of the HFD-control group: *** *P* < 0·001.

As shown in Fig. 3(a), after 1 week under the diets, the fasting glycaemia of the HFD-control group (159 (sd 3·9) mg/dl; 8·8 (sd 0·2) mmol/l) was significantly higher than that of the ND-control group (109 (sd 3·6) mg/dl; 6·0 (sd 0·2) mmol/l; *P* < 0·001). This difference then stayed relatively constant during the rest of the study.

Chronic oral administration of the CAE significantly reduced blood glucose levels compared with the HFD-control group treated with water. This effect was significant from the second week of treatment onwards, and lasted throughout the study (with a mean 44·8 % reduction of hyperglycaemia induced by the HFD; *P* < 0·001).

By the end of the study, fasting insulinaemia was also significantly increased by the HFD. Indeed, the blood insulin level of the HFD-control group reached 4·29 (sd 0·08) ng/ml, whereas that of the ND-control group reached 1·65 (sd 0·14) ng/ml (Fig. 3(b)). In line with the fasting glycaemia and fasting insulinaemia increases, the insulin resistance index (HOMA-IR) was four times higher in the HFD-control mice compared with the ND-control mice (Fig. 3(c)).

As presented in Fig. 3(b), treatment of the mice with the CAE prevented the increase in fasting blood insulin level induced by the HFD. Insulinaemia reached 3·54 (sd 0·35) and 4·29 (sd 0·08) ng/ml for HFD-CAE mice and HFD-control mice, respectively. This effect was close to being statistically significant (*P* = 0·051). Therefore, according to the HOMA-IR insulin resistance index, the CAE significantly reduced insulin resistance induced by the HFD in DIO mice by 39·5 % (*P* < 0·01; Fig. 3(c)).

### Curative effects of the cashew apple extract on diet-induced obesity mice

#### Curative effects of the cashew apple extract on body weight, fat storage and energy consumption

**Fig. 4.**
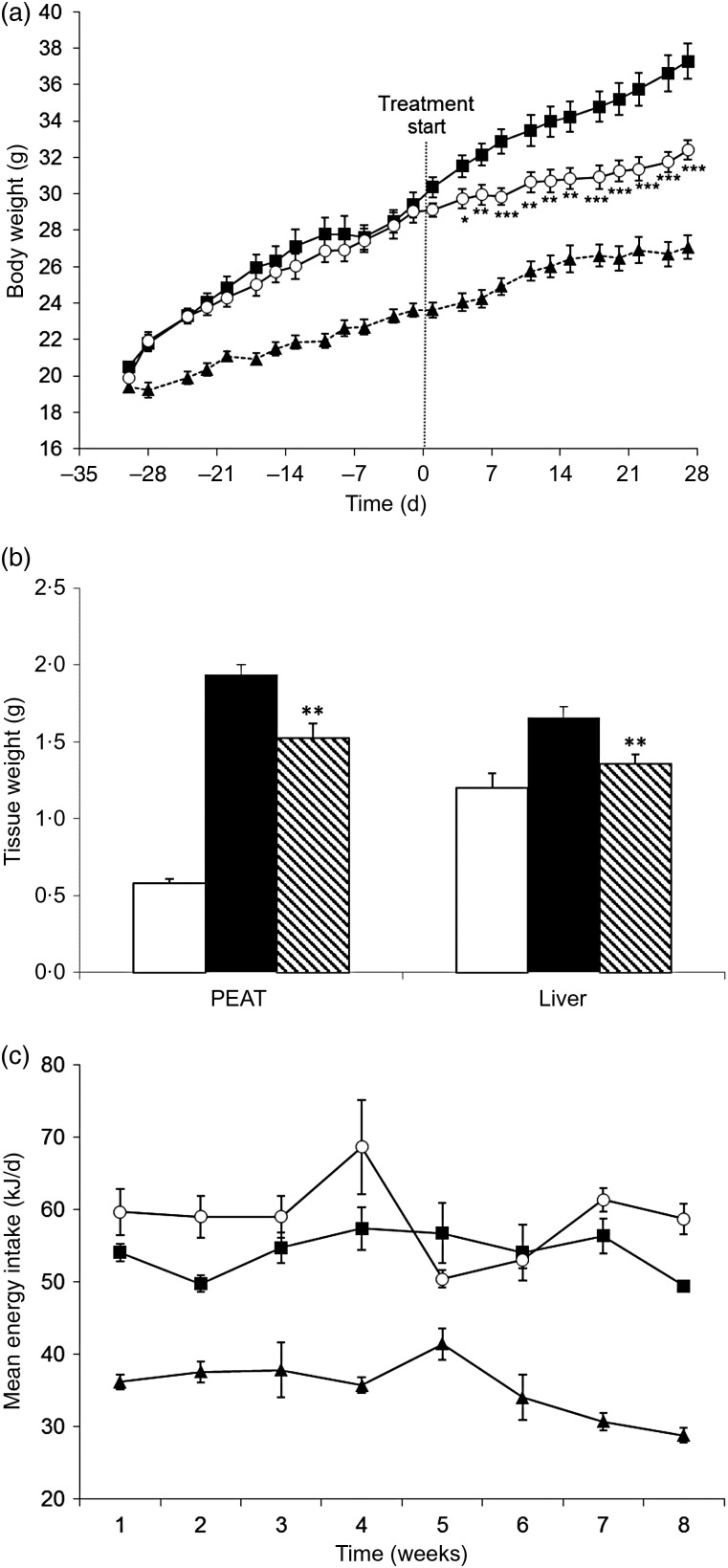
Curative effects of the cashew apple extract (CAE) on (a) body weight, (b) peri-epididymal adipose tissue (PEAT) and liver weights, and (c) energy intake in diet-induced obesity mice. (a) and (c): --▲--, Normal diet-control (ND-control); –■–, high-fat diet-control (HFD-control); –○–, CAE (200 mg/kg body weight). (b): □, ND-control; ■, HFD-control; \\\, CAE (200 mg/kg body weight). Values are means (*n* 9), with standard errors represented by vertical bars. Mean value was significantly different from that of the HFD-control group: * *P* < 0·05, ** *P* < 0·01, *** *P* < 0·001.

After 4 weeks under the diets, mice submitted to the HFD presented a significant difference in body weight compared with mice submitted to the ND (*P* < 0·001), showing that obesity was well established before the beginning of the treatment. The HFD-control group reached 30·4 (sd 0·5) g body weight, whereas the ND-control group reached 23·6 (sd 0·4) g (Fig. 4(a)).

As shown in Fig. 4(a), the treatment with the CAE reduced the body-weight gain of the mice. This effect was significant from the fourth day of treatment onwards and lasted throughout the treatment period. At the end of the study, the CAE-treated group reached 32·4 (sd 0·6) g body weight whereas water-treated mice (HFD-control) reached 37·3 (sd 1) g. Mice orally treated with the CAE gained 4·5 g (57·2 %) less weight compared with HFD-control mice (*P* < 0·001). In line with the observed decrease in body-weight gain, the CAE reduced fat storage induced by the HFD in the PEAT by 30·3 % (*P* < 0·01). The PEAT weight of CAE-treated mice was 1·52 (sd 0·1) g, compared with 1·93 (sd 0·1) and 0·58 (sd 0·03) g for HFD-control mice and ND-control mice, respectively (Fig. 4(b)). The CAE reduced fat storage induced by the HFD in the liver by 65·9 % (*P* < 0·01). The liver weight of CAE-treated mice was 1·36 (sd 0·06) g, compared with 1·66 (sd 0·07) and 1·20 (sd 0·07) g for HFD-control mice and ND-control mice, respectively (Fig. 4(b)).

As for energy consumption, mice under the HFD consumed more energy than mice under the ND, but no significant differences were observed between the HFD-control and HFD-CAE groups (Fig. 4(c)).

#### Curative effects of the cashew apple extract on glycaemia, insulinaemia and insulin resistance

After 4 weeks under the diets, fasting glycaemia of the mice submitted to the HFD (173 (sd 1·5) mg/dl; 9·6 (sd 0·1) mmol/l) was significantly higher (*P* < 0·001) than fasting glycaemia of the mice submitted to the ND (115 (sd 2·1) mg/dl; 6·4 (sd 0·1) mmol/l). These results confirm the fact that the mice under the HFD were hyperglycaemic at the onset of the treatment (Fig. 4(a)).

**Fig. 5.**
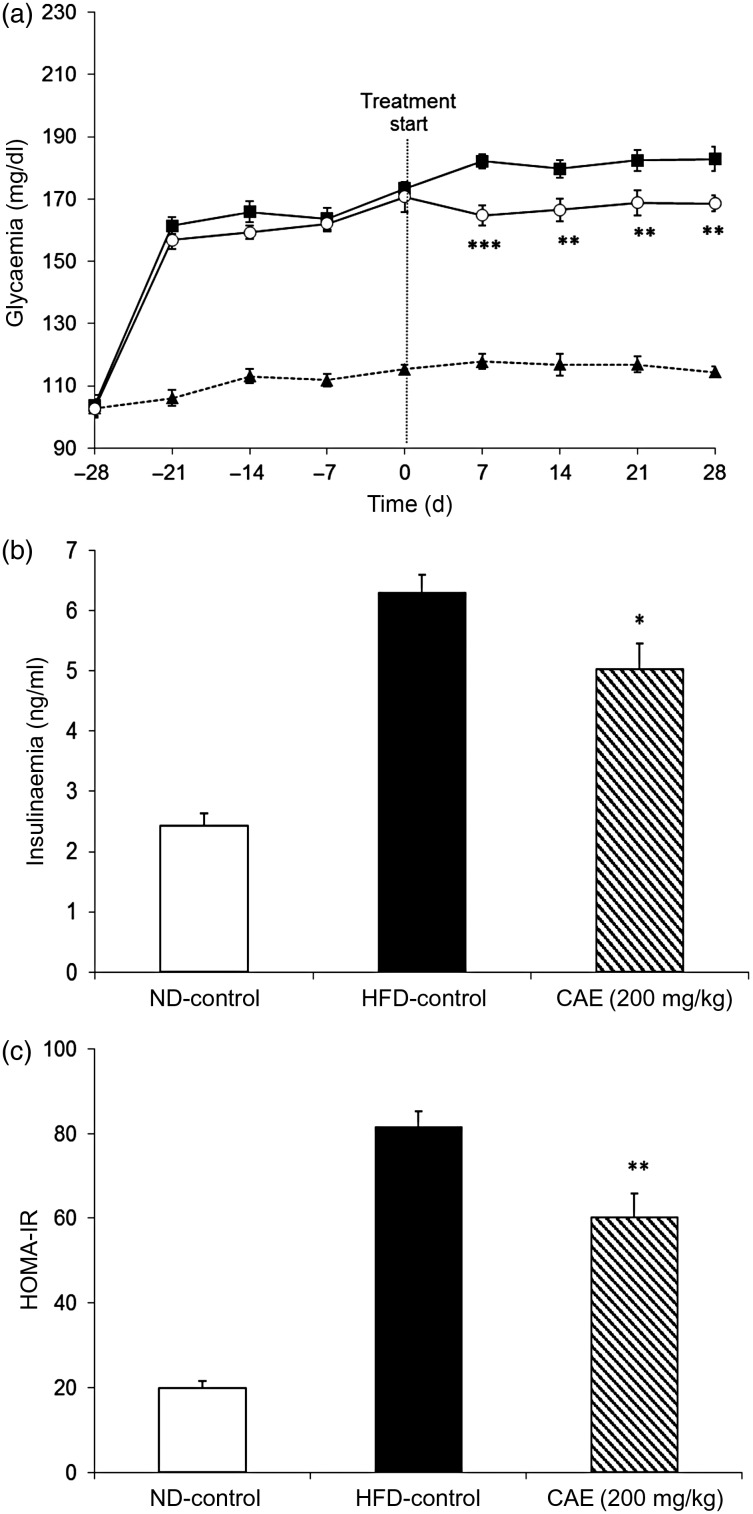
Curative effect of the cashew apple extract (CAE) on (a) glycaemia, (b) insulinaemia and (c) insulin resistance (homeostasis model assessment-insulin resistance; HOMA-IR) in diet-induced obesity mice. (a): --▲--, Normal diet-control (ND-control); –■–, high-fat diet-control (HFD-control); –○–, CAE (200 mg/kg body weight). To convert glycaemia from mg/dl to mmol/l, multiply by 0·0555. Values are means (*n* 9), with standard errors represented by vertical bars. Mean value was significantly different from that of the HFD-control group: * *P* < 0·05, ** *P* < 0·01, *** *P* < 0·001.

As of the first week of treatment, and throughout the study, the CAE significantly reduced the hyperglycaemia induced by the HFD in mice by a mean of 22·4 % (*P* < 0·05 to *P* < 0·001, depending on the time point; Fig. 5(a)). At the end of the protocol, insulinaemia and insulin resistance were also reduced by the treatment with the CAE. The blood insulin level of CAE-treated mice reached 5·03 (sd 0·43) ng/ml, whereas the levels for the HFD-control group reached 6·30 (sd 0·29) ng/ml and the levels for the ND-control group reached 2·43 (sd 0·20) ng/ml (Fig. 5(b)). According to the HOMA-IR insulin resistance index, the CAE reduced insulin resistance induced by HFD in DIO mice by 34·4 % (*P* < 0·01; Fig. 5(c)).

## Discussion

The metabolic syndrome is a pre-disease condition without any known remedy other than improving diet and increasing physical exercise. Overweight and obesity are common characteristics of the metabolic syndrome, increasing the risk of type 2 diabetes occurring. With our objective to identify active plant extracts for the prevention of overweight and obesity in mind, we screened various plant raw materials for that purpose. As part of our approach we gathered various agricultural by-products as they constitute sustainable materials, for some very rich in polyphenols and other potentially active molecules. The cashew apple is a poorly valued by-product of the cashew nut industry which is available in significant volumes. It was selected for our research amongst other raw material candidates. From here, we developed and optimised a specific CAE which was tested in a DIO animal model.

The C57BL/6 DIO mouse model provides a well-recognised nutritional model of obesity and pre-diabetes. It is widely used to test drugs as well as complex plant extracts^(^^33^^,^^34^^)^. For our work, we used two different designs: a ‘prevention’ design so as to evaluate the ability of the extract to prevent the development of obesity, hyperglycaemia and insulin resistance, and a ‘curative’ design to test its capacity to reverse an established disease state.

The daily oral intake of 200 mg/kg body weight of the CAE, in both the preventive and the curative study, resulted in a significant body-weight-gain reduction of the mice submitted to a HFD. This reduction in body-weight gain was at least partly due to a decrease in the peri-epididymal (perivisceral) adipose tissue mass. Increase in perivisceral fat is recognised to be associated with, or to be a strong risk factor for, insulin resistance and the metabolic syndrome^(^^35^^)^. Hence the prevention of this tissue's increase, as it has been observed in mice treated with CAE, should contribute to the improvement in insulin resistance. Next to that, fat was also stored under the form of TAG in the liver (hepatic steatosis), resulting from the imbalance between energy intake and expenditure, combined with higher insulin resistance, oxidative stress and inflammation^(^^36^^)^. Liver weight and TAG content analysis performed in the preventive study showed that CAE treatment also prevented fat storage in the liver and, consequently, should improve liver functions. It is interesting to note that the CAE reduced body-weight gain without affecting food consumption as the CAE-treated and water-treated animals ate the same amount of HFD and then the same level of energy. Hence the CAE may reduce fat storage by reducing fat digestion and absorption, increasing fat metabolism, energy expenditure or thermogenesis. Further investigations are needed to clarify the CAE mechanism of action.

Next to its effect on body weight, CAE supplementation also reduced hyperglycaemia, hyperinsulinaemia and insulin resistance of DIO mice in both study designs. The reduction in body weight and the reduction in fat storage in the perivisceral adipose tissue as well as in the liver was probably linked to the improvement of insulin sensitivity and glycaemic control as it is recognised that even a small reduction in body weight can improve insulin action^(^^37^^)^. However, we cannot exclude a direct action of the CAE on insulin resistance at this stage of analysis.

The CAE is enriched in flavonoids and more precisely in myricetin and quercetin derivatives. Flavonoids are plant polyphenols that are recognised as effective antioxidants^(^^38^^)^ and may protect against several chronic diseases^(^^39^^)^. Among flavonoids, diets enriched in myricetin and quercetin tend to lower the risk of developing type 2 diabetes^(^^39^^)^. Choi *et al*. showed that myricetin supplementation had a protective effect on obesity and insulin resistance in C57BL/6 mice fed with a high-fat and high-sucrose diet^(^^40^^)^. Similar results were obtained by Henagan *et al*. by using quercetin and a quercetin-rich onion extract in the same mice submitted to a HFD^(^^41^^)^. *In vitro* studies indicated that myricetin and quercetin flavonoids may inhibit intestinal glucose transporters^(^^42^^)^ and/or glucose uptake in adipocytes^(^^43^^)^. Combined, these data suggest that myricetin and quercetin derivatives contained in plant extracts such as CAE may participate in the effect of the extract on DIO mice. Also, a poorly described cinnamic acid derivative recently identified in cashew apple^(^^22^^)^, 1-*O-trans*-cinnamoyl-β-d-glucopyranose, is present in the CAE at almost the same amount as quercetin derivatives. The role of this phytochemical in the CAE activity observed in this study is not known. At last, the phenolic and non-phenolic compounds may also participate in the protective effect of the CAE against DIO and insulin resistance.

## Conclusion

We reported here for the first time that the oral administration of a water-alcoholic CAE (Cashewin^™^) can reduce body-weight gain, fat storage, hyperglycaemia, hyperinsulinaemia and insulin resistance in DIO mice. This has been evidenced in both a preventive and a curative *in vivo* study design. Further investigations have to be carried out to identify more specifically which cashew apple molecules, and through which mechanisms of action, are responsible for achieving these protective effects.
